# The role of chemerin, elafin, and visfatin in the pathogenesis of atopic dermatitis

**DOI:** 10.3389/fimmu.2025.1628163

**Published:** 2025-09-03

**Authors:** Mateusz Matwiejuk, Agnieszka Kulczyńska-Przybik, Hanna Myśliwiec, Agnieszka Mikłosz, Adrian Chabowski, Barbara Mroczko, Iwona Flisiak

**Affiliations:** ^1^ Department of Dermatology and Venereology, Medical University of Bialystok, Bialystok, Poland; ^2^ Department of Neurodegeneration Diagnostics, Medical University of Bialystok, Bialystok, Poland; ^3^ Department of Physiology, Medical University of Bialystok, Bialystok, Poland

**Keywords:** atopic dermatitis, chemerin, elafin, visfatin, NAMPT

## Abstract

Atopic Dermatitis is a chronic skin condition characterized by inflammation and itching. It has a genetic component, but environmental factors also play a significant role. The immune system is overactive, leading to an abnormal inflammatory response. Literature data indicate that numerous proteins contribute to the development and progression of atopic dermatitis, like antimicrobial peptides, alarmins, autoantigens, cytokines, growth factors, and proteases. To synthesize current knowledge and identify the most promising contributors of AD pathogenesis a literature search was conducted using PubMed (1990–present), Google Scholar, and Embase, has been performed appropriate search terms. This narrative review summarizes the current knowledge on how elafin, chemerin, and nicotinamide phosphoribosyltransferase (visfatin/NAMPT) contribute to the pathophysiology of skin inflammation in atopic dermatitis. Recent discoveries have highlighted the importance of these proteins as important players in the functioning of the epidermal barrier. Importantly, some proteins exert anti-inflammatory effects (e.g., elafin), some pro-inflammatory effects, such as visfatin/NAMPT or chemerin, which exhibits both pro- and anti-inflammatory properties. This makes them intriguing candidates for modulating the complex inflammatory processes associated with atopic dermatitis. A deeper understanding of the role of these proteins may provide a basis for the development of appropriate treatments for atopic dermatitis. However, knowledge about the importance of these proteins in the pathological mechanisms of atopic dermatitis is still limited.

## 1 Introduction

### 1.1 Epidemiology

Atopic dermatitis (AD), also known as an atopic eczema, is the most common chronic, allergic, inflammatory skin disease worldwide (1). The significant burden that AD places on both healthcare funding and the quality of life of patients prompts a deeper understanding of the pathomechanisms of this disease (2). In 2021, the global number of pediatric AD cases reached 72.4 million, a 6.2% increase from 2000 (3). In adults, the prevalence of AD estimated between 3.4% in Israel to 33.7% in Thailand. Additionally, it was revealed that the prevalence was generally higher in females compared to males (4).

### 1.2 Symptoms

The “atopic march” describes the progression of atopic diseases, often starting with AD in infancy or early childhood, followed by the development of food allergies, allergic rhinitis (hay fever), and asthma (5). Eczematous skin, the type of inflammation seen in AD, is often described as redness, swelling, and oozing. The skin rashes and lesions appear in similar patterns on both sides of the body. Importantly, skin lesions in AD are characterized by age-dependent locations. The area’s most commonly affected by AD change with age: in infants these are: face, scalp, extensor surfaces of extremities (elbows, knees); in children: flexural areas (creases of elbows, knees, neck); and in adults: hands, feet, eyelids (6). Hanifin and Rajka Score are set of various criteria, which is a well-known and widely used method for diagnosing AD. To recognize AD is required to have three of four major criteria and three of twenty-three minor criteria (7). Moreover, AD is often associated with significant pruritus, which can significantly impact the quality of life of those who suffer from it. Scratching can further worsen the condition and impede healing (8).

### 1.3 Pathophysiology

The pathogenesis of AD comprises a complex interplay of factors, including impairments in both the innate and adaptive immune responses (9). These immune dysfunctions, combined with a compromised skin barrier, contribute to the inflammation and skin lesions characteristic of the condition. When the skin barrier is compromised, keratinocytes (the main cells of the epidermis) release increased amounts of certain cytokines, including thymic stromal lymphopoietin (TSLP) and interleukins such as IL-25 and IL-33. These cytokines play a role in activating the immune system, particularly T helper 2 (Th2) cells, and are involved in promoting type 2 immune responses Particularly TSLP initiates a cascade of events. Firstly, TSLP stimulates dendritic cells to express OX40L, which then interacts with OX40 on T cells, promoting Th2 cell differentiation. Secondly, this Th2 cell activation by TSLP, leads to the production of inflammatory cytokines like IL-4, IL-5, and IL-13, characteristic of type 2 inflammation (10). Furthermore, filaggrin (FLG) is a protein crucial for maintaining the skin’s barrier function. Mutations in the FLG gene are strongly associated with the development of AD. These mutations disrupt the skin barrier, leading to: an increased transepidermal water loss (TEWL) (presented as a dry, cracked skin); an increased penetration of allergens and irritants: (showed as triggering inflammation); and dysregulation of the immune system (11). Moreover, higher FLG mRNA levels were found in patients with severe AD compared to patients with moderate AD, suggesting that the body attempts to compensate for the weakened skin barrier by increasing FLG production (12).

**Figure 1:**
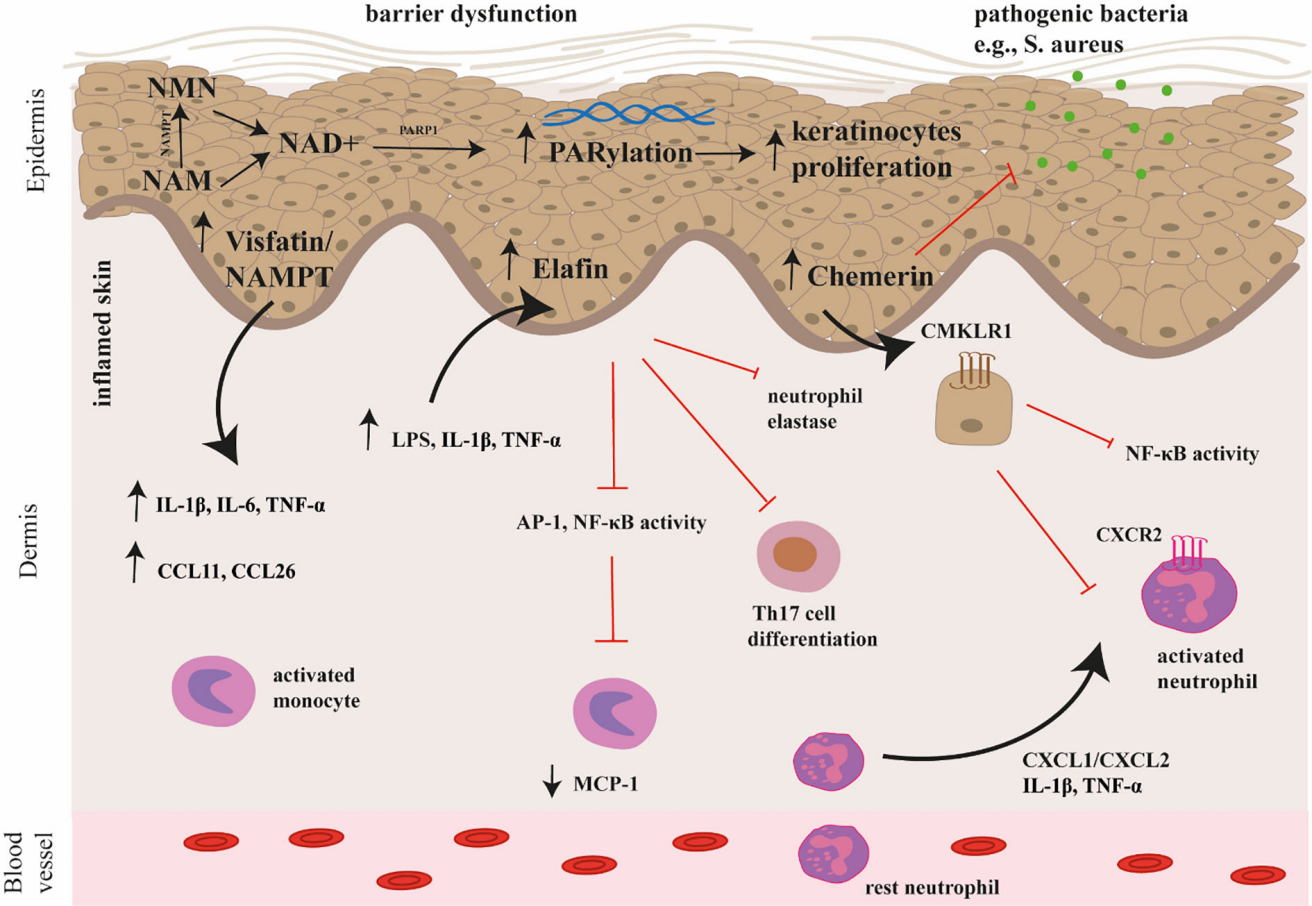
The potential mechanism of chemerin, elafin, and visfatin/NAMPT action in the pathogenesis of AD. Chemerin exerts antimicrobial activity and immunomodulatory effects through interactions with the CMKLR1 receptor. In particular, chemerin attracts neutrophils to leave inflammatory sites by interacting with CCRL2, which may participate in the spread of inflammation. Elafin reduces skin inflammation and tissue damage by inhibiting the activity of transcription factors (AP-1, NF-κB) involved in the production of pro-inflammatory cytokines and by inhibiting the activity of neutrophil elastase. Moreover, it affects the differentiation and function of Th17 cells. Visfatin/NAMPT is the rate limiting enzyme in the NAD^+^ salvage pathway, converting NAM into NMN, which is then converted to NAD^+^. Subsequently, poly(ADP-ribose) (PAR) polymerases (PARP) consume NAD+ during the PARylation process. Overactivation of PARP mediates skin inflammation and promotes keratinocyte proliferation. Visfatin/NAMPT increases the secretion of pro-inflammatory cytokines such as IL-1β, IL-6, TNF-α, as well as chemokines (e.g., CCL11, CCL26). AP-1, activator protein 1; CCL11, CC Motif Chemokine Ligand 11; CCL26, CC Motif Chemokine Ligand 26; CCRL2, C-C Chemokine receptor-like 2; CMKLR1, Chemerin Chemokine-Like Receptor 1; IL-1β, interleukin 1β, IL-6, interleukin 6; NAD+, nicotinamide adenine dinucleotide; NAM, nicotinamide; NMN, nicotinamide mononucleotide; PARP, poly(ADP-ribose) (PAR) polymerase; TNF-α, tumor necrosis factor α.

Recent studies have shown that elafin (13), chemerin (14) and nicotinamide phosphoribosyltransferase (NAMPT)/visfatin (15) may be potential key players in the pathogenesis of AD. The potential mechanism of their action in AD is shown on the **Figure 1**. Briefly, elafin is a protein that acts as a serine protease inhibitor and is involved in the body’s defense against inflammation. Chemerin is involved in immune cell recruitment and inflammation, which are also characteristic of AD. In turn, NAMPT, also known as visfatin, is an enzyme involved in NAD+ biosynthesis and has pro-inflammatory properties. The link between these proteins and AD suggests that they may be novel targets for therapeutic intervention.

A comprehensive understanding of the role of chemerin, elafin, and visfatin in the pathogenesis of atopic dermatitis (AD) is essential for the development of novel and more effective therapeutic strategies. Although these proteins have been increasingly studied in recent years, their specific functions in AD remain incompletely understood, with some studies reporting conflicting findings. This narrative review aims to synthesize the current evidence regarding their involvement in AD, identify gaps in the existing knowledge, and propose future research directions. By clarifying the roles of these mediators in AD, this review seeks to offer timely and clinically relevant insights that may inform the development of targeted treatments and ultimately improve patient outcomes. While previous reviews have focused on single mediators, our study integrates evidence on three structurally and functionally distinct proteins implicated in AD. A literature search was conducted using PubMed (1990–present), Google Scholar, and Embase, employing appropriate search terms.

## 2 Discussion

### 2.1 Elafin – an overview

Elafin and its precursor, trappin-2, are potent inhibitors of human serine proteases, specifically those belonging to the chelonianin family (16). The protein is an epithelial host-defense protein that is absent in normal skin, but is significantly upregulated in keratinocytes in inflamed skin (17). Elafin, by inhibiting excessive protease activity, plays a crucial role in protecting the skin’s epidermal structure and integrity. This is crucial for maintaining the skin’s barrier function and preventing damage. Therefore, elafin may facilitate epidermal repair and regeneration after injury. Furthermore, elafin may influence the migration of polymorphonuclear leukocytes (PMNs), a type of white blood cell involved in inflammation and wound healing. By modulating PMN migration, elafin helps to ensure a controlled and effective inflammatory response (18). Elafin has a positive charge, which is important for its interactions with other molecules and its function. Proinflammatory cytokines like IL-1 and TNF-α stimulate the production of elafin, indicating its role in the inflammatory response (19). In summary, elafin has been shown to possess significant antimicrobial properties against bacteria, viruses, parasites, and fungi (20).

Elafin inhibits lipopolysaccharide (LPS)-induced production of monocyte chemotactic protein 1 (MCP-1) in monocytes by blocking the activation of activatior protein (AP-1) and NF-κB, which are key transcription factors in pro-inflammatory cytokine production, including those involved in Th17 differentiation. The suppression of AP-1 and NF-κB signaling could potentially lead to a downregulation of signals that promote Th17 differentiation and subsequent cytokine production (**Figure 1**) (21, 22).

#### 2.1.1 Elafin’s role in the pathogenesis of atopic dermatitis

Current studies indicate that elafin could be a pivotal indicator of the AD (**Table 1**). Brunner et al. (23) found that in children with AD, blood levels of markers associated with Th2 (Chemokine (C-C motif) ligand 13 (CCL13), Chemokine (C-C motif) ligand 22 (CCL22)) and Th17 (elafin) were significantly higher compared to healthy children. The study found only weak correlations between BMI and inflammatory markers (elafin, IL-16, and IL-2RA). This suggests that while obesity contributes to inflammation, its impact on the specific inflammatory pathways involved in pediatric AD may be limited (23) (**Table 1**).

**Table 1:** Summary of the studies on elafin’s role in atopic dermatitis.

Author	Year	Population	Key observation
Elafin in atopic dermatitis			
Brunner et al. (23)	2019	N2 – children patients with AD N3–61 adult patients with AD	A weak correlation was found between BMI and some inflammatory markers (elafin).
Esaki et al. (24)	2016	N1–14 healthy children N2–19 children with AD N3–8 healthy adults N4–15 adults with AD	The skin of children with AD exhibited significantly higher levels of Th17-related cytokines and antimicrobial peptides (like elafin) compared to the skin of adults with AD.
Facheris et al. (25)	2023	N1–15 healthy patients N2–15 AOAD patients N3–15 POAD patients	POAD exhibited higher levels of inflammation, where a higher level of elafin was observed, in their affected skin compared to those with AOAD.
Brunner et al. (13)	2017	N1–18 healthy patients N2–59 patients with AD	Elafin is one of the key inflammatory markers associated with severity of AD correlations were observed in both affected and unaffected skin of AD patients.
Gittler et al. (26)	2012	N – 17 patients with AD	During AD transitions from acute to chronic phases, there were significant increases in the expression of genes regulated by elafin.
Lancto et al. (27)	2013	N1–16 healthy dogs N2–13 dogs with AD	Deficiency in antimicrobial peptides, particularly elafin, may contribute to the development and progression of AD and other inflammatory skin conditions in dogs
Kamsteeg et al. (28)	2010	N2–16 patients with ACD N3–6 patients with AD N4–6 patients with psotiasis	Expression of elafin in skin of patients with AD and ACD was variable and less intense in comparison to those with psoriasis
Guttman-Yassky et al. (29)	2008	N1–15 healthy people N2–18 patients with AD N3–18 patients with psoriasis	lower expression of elafin in AD skin lesions, compared to psoriatic lesions
Pavel et al. (30)	2019	36 patients with AD	ASN002 downregulates elafin, which is also involved in skin barrier function and inflammation in patients with AD.
Jensen et al. (31)	2012	15 patients with AD	Topical treatment resulted in a reduction of elafin (inflammatory marker) expression in the skin of AD patients.
Khattri et al. (32)	2017	N1–17 AD patients with placebo N2–16 AD patients with ustekinumab	Ustekinumab effectively inhibits the activity of both Th1 and Th17 immune cells by blocking the signaling pathways involving IL-12 and IL-23, like decrease of elafin.
Khattri et al. (33)	2014	N – 19 patients with AD	The ciclosporin A treatment led to a suppression of genes include those involved in producing proteins like elafin.
Czarnowicki et al. (34)	2016	N1–36 healthy patients N2–13 patients with AD	Petrolatum application significantly increased the expression of elafin. This suggests that petrolatum may stimulate the skin’s natural defenses against infection.

CCL7, chemokine C-C motif ligand 7; BMI, body mass index; POAD, pediatric-onset AD; AOAD, adult-onset atopic dermatitis; JAK, Janus Kinase Inhibitor; SYK, Spleen Tyrosine Kinase; CCL13, chemokine C-C motif ligand 13; CCL22, Chemokine C-C motif ligand 22; CCL20, chemokine ligand 20; LCN2, lipocalin 2; S100A7-9, S100 Calcium Binding Protein A7-9; CCL17, CC chemokine ligand 17; S100A8, S100 Calcium Binding Protein A8; S100A9, S100 Calcium Binding Protein A9; SLPI, secretory leukocyte proteinase inhibitor; CBD-1, canine beta-defensin 1; CBD-103, canine beta-defensin 103; CBD-122, canine beta-defensin 122; ACD, allergic contact dermatitis.

Most studies report increased levels of elafin in AD; however, there is variation depending on disease severity and model used.

The study by Esaki et al. (24) found that children with AD exhibited significantly higher levels of Th17-related cytokines and antimicrobial peptides (like elafin) compared to adults with AD. Importantly, similar to the studies mentioned above, this study observed strong correlations between disease activity scores and markers associated with Th2 and Th17 pathways (including elafin). Precisely, AD severity was strongly associated with IL-17 (cytokine produced by Th17)-related elafin marker. This suggests that these immune pathways play a significant role in driving the severity of the disease (24) (**Table 1**).

Facheris et al. (25) found that individuals with pediatric-onset AD (POAD) persisting into adulthood have higher levels of inflammation and elafin in their affected skin compared to those with adult-onset atopic dermatitis (AOAD). These differences between POAD and AOAD regarding these markers were statistically significant, with a p-value less than 0.05 (25) (**Table 1**).

In line with previous findings, Brunner et al. (13) investigated the relationship between inflammatory markers and AD severity They found that serum levels of elafin are associated on the extent of AD skin disease. Importantly, these correlations were observed in both affected and unaffected skin of AD patients. On the other side, elafin was also linked to atherosclerosis development alongside other inflammatory markers like E-selectin, Chemokine CC motif ligand 7 (CCL7), IL16. The study also established a strong link between elafin and AD severity, but interestingly, no correlation was found between elafin levels and body mass index (BMI) (13) (**Table 1**).

Gittler et al. (26) revealed that Th2 and Th22 immune responses are progressively activated as AD transitions from acute to chronic phases. The study found a positive correlation between the SCORAD index and the expression of IL-22 mRNA in acute disease. This suggests that IL-22 may play a significant role in the severity of acute AD. While small increases in Th17-related cytokines (IL-17, IL-23p19, and IL-23p40) were observed, there were significant increases in the expression of genes regulated by elafin, IL-17, such as chemokine ligand 20 (CCL20), and lipocalin 2 (LCN2). This suggests that the Th17 pathway is also involved in acute AD, although to a lesser extent than Th2 and Th22 (26) (**Table 1**).

On the other hand, Lancto et al. (27) observed significantly lower transcript levels for elafin, secretory leukocyte proteinase inhibitor (SLPI), canine beta-defensin 1 (CBD-1), canine beta-defensin 103 (CBD-103), and canine beta-defensin 122 (CBD-122) in both lesional and non-lesional skin of dogs with AD compared to healthy skin. Significantly higher elafin expression was observed on the inner thigh compared to the scapula, axilla, and forehead. These findings highlighted that a deficiency in antimicrobial peptides, particularly elafin and SLPI, may contribute to the development and progression of atopic dermatitis and other inflammatory skin conditions in dogs. This could potentially lead to increased susceptibility to skin infections and exacerbation of the inflammatory response (27) (**Table 1**).

In contrast to the above-mentioned studies, Kamsteeg et al. (28) showed that elafin expression in the skin of patients with AD and allergic contact dermatitis (ACD) was variable and less intense compared to those with psoriasis. As expected, no elafin was detected in healthy skin (28) (**Table 1**).

Guttman-Yassky et al. (29) found that elafin, S100A7, S100A9, and cystatin A mRNAs were highly expressed in psoriasis skin lesions. While these genes were also expressed at lower levels in atopic dermatitis (AD) lesions compared to psoriasis, their expression was still higher in AD lesions than in healthy skin, suggesting a potential role in AD inflammation, albeit less prominent than in psoriasis (29) (**Table 1**).

Pavel et al. (30) showed that ASN002 represents a potential new treatment option for AD directly targeting the underlying inflammatory mechanisms of the disease. ASN002 is an oral medication that inhibits two key signaling pathways: Janus Kinase (JAK) and Spleen Tyrosine Kinase (SYK). In addition, its ability to modulate multiple signaling pathways also includes TH2, TH22, TH17, and TH1 pathways, all of which play a crucial role in AD (30) (**Table 1**).

The study by Jensen et al. (31) demonstrated that both pimecrolimus and betamethasone valerate treatments led to a decrease in elafin expression in the skin of patients with atopic dermatitis (AD). This suggests that both medications effectively reduce inflammation in AD (31) (**Table 1**).

Khattri et al. (32) suggested that ustekinumab, a biological drug, effectively targets and suppresses specific immune pathways involved in Atopic Dermatitis (AD) by inhibiting the activity of Th1 and Th17 immune cells. This inhibition is achieved by blocking the signaling pathways involving IL-12 and IL-23, potentially leading to a reduction in inflammation in conditions where these cells play a significant role (32) (**Table 1**).

Khattri et al. (33) observed that ciclosporin A treatment suppressed genes associated with Th2, Th22, and some Th17 immune responses. These genes include those involved in producing proteins like elafin, S100 Calcium Binding Protein A7-9 (S100A7-9), CC chemokine ligand 17 (CCL17), and S100As. In addition, this suppression of immune responses was correlated with reduced epidermal hyperplasia (skin thickening) and overall clinical improvement in AD patients. The study also noted that these changes in gene expression occurred in both lesional and non-lesional skin (33) (**Table 1**).

Czarnowicki et al. (34) described a study investigating the effects of petrolatum on AD skin. Its application significantly increased the expression of various antimicrobial peptides (S100 Calcium Binding Protein A8 (S100A8), S100 Calcium Binding Protein A9 (S100A9), CCL20, elafin, lipocalin 2, human β-defensin 2) and innate immune genes (IL-6, IL-8, and IL-1β) in the skin of patients with AD. This suggests that petrolatum may stimulate the skin’s natural defenses against infection. Moreover, petrolatum also stimulated the expression of key barrier differentiation markers (filaggrin and loricrin), which are essential for maintaining a healthy skin barrier. Additionally, it increased the thickness of the stratum corneum (the outermost layer of the skin), further enhancing the skin’s barrier function (34) (**Table 1**).

### 2.2 Chemerin – an overview

In 1997, chemerin was identified as a tazarotene-induced gene-2 (TIG-2) in psoriatic skin (35), Chemerin is initially secreted in an inactive precursor form known as prochemerin (36). Moreover, chemerin and its receptors (chemokine-like receptor 1 (CMKLR1) (36), G protein-coupled receptor 1 (GPR1) (37), C-C chemokine receptor-like 2 (CCRL2) (38) significantly contribute to various biological processes, like adipogenesis (39), osteoclastogenesis (40), angiogenesis (41), and skin inflammation (42). Regarding the role of chemerin in the skin, human keratinocytes cultured in the laboratory respond to specific microbial signals by altering the expression of chemerin and its receptors. Furthermore, chemerin has been found to be essential for the efficient clearance of bacteria in a skin infection model (43).

Chemerin has dual, or “chimeric,” effects on inflammation: pro-inflammatory via activation of the ChemR23 receptor or anti-inflammatory via CMKLR1. For example, activation of CMKLR1 has been linked to suppression of the transcription factor NF-κB and a shift of the macrophage phenotype toward a less inflammatory state. Chemerin has a chemotactic effect on immune cells, promoting cell migration in inflammatory conditions; on the other hand, it inhibits the action of pro-inflammatory cytokines such as interleukin 6 (IL-6) and tumor necrosis factor α (TNF-α), exerting anti-inflammatory effects. However, the precise balance of these effects in AD is still under investigation (44).

#### 2.2.1 Chemerin’s role in the pathogenesis of atopic dermatitis

Literature data indicated that chemerin is engaged in pathological mechanisms underlying some skin diseases, including AD. Xiao et al. (14) discussed the role of IL-13 and toll-like receptor 2 (TLR2) in neurogenic inflammation, particularly in AD and itch. Pam3CSK4 (a TLR2 agonist) enhances the effects of IL-13 in cultured sensory neurons. This includes increased calcium signaling, a higher number of responding neurons, and the release of pro-inflammatory cytokines. In sensory neurons (mDRGs), pre-treatment with Pam3CSK4 significantly increased the secretion of cytokines (chemerin, TNFα, CCL17, and CCL20) in response to IL-13. This enhancement was significantly reduced in neurons with reduced IL-13Rα2 expression (achieved through IL-13Rα2 knockdown). Furthermore, these findings suggest that the interplay between IL-13 and TLR2 signaling pathways is crucial in driving neurogenic inflammation in AD. Summing up, targeting both IL-13-IL-13Rα2 and TLR2-IL-13Rα2 pathways could be a promising therapeutic strategy for treating AD and associated itch (14) (**Table 2**).

**Table 2:** Summary of the studies on chemerin’s role in atopic dermatitis.

Author	Year	Population	Key observation
Chemerin in atopic dermatitis			
Xiao et al. (14)	2021	C57BL/6 female mice	In sensory neurons (mDRGs), pre-treatment with Pam3CSK4 significantly increased the release of cytokines like chemerin in response to IL-13. It suggests that the interplay between IL-13 and TLR2 signaling pathways is crucial in driving neurogenic inflammation in AD.
Albanesi et al. (45)	2009	N1–10 people N2–5 patients with AD	No association between the presence of chemerin-expressing cells and pDCs or neutrophils in AD skin.

N1, control group; N2, study group; TLR2, toll-like receptor 2; TNFα, tumor necrosis factor alpha; pDCs, plasmacytoid dendritic cells.

Albanesi et al. (45) presented data on the distribution of chemerin and its potential role in AD. Very few chemerin-expressing cells were found in the dermis of AD skin lesions. This contrasts with findings in psoriasis, where chemerin plays a role in early lesion development. AD lesions showed minimal infiltration of plasmacytoid dendritic cells (pDCs). Importantly, there was no association between the presence of chemerin-expressing cells and pDCs or neutrophils in AD skin. Taken together these results suggest that the chemerin pathway may not play a significant role in the pathogenesis of AD, unlike in the pathogenesis of psoriasis (45) (**Table 2**).

### 2.3 Visfatin/NAMPT – an overview

Visfatin/NAMPT is a protein also known as pre-B-cell colony-enhancing factor (PBEF) and nicotinamide phosphoribosyltransferase (NAMPT), It is primarily secreted by visceral adipose tissue (46). Visfatin/NAMPT has a dual role, acting as both an intracellular enzyme and an extracellular protein. Inside cells, it acts as an enzyme that converts nicotinamide (NAM) into nicotinamide mononucleotide (NMN). However, in both intracellular and extracellular contexts, the produced NMN is then converted to nicotinamide adenine dinucleotide (NAD) by the enzyme nicotinamide/nicotinic acid mononucleotide adenyltransferase (NMNAT). NAD is a vital coenzyme involved in various cellular processes, including energy production, cellular signaling, and DNA repair (47). Visfatin/NAMPT enhances the production of pro-inflammatory cytokines, specifically IL-6 and IL-1β, in both human monocytes (48) and endothelial cells (49), supporting the classification of visfatin/NAMPT as an inflammatory adipokine.

#### 2.3.1 Visfatin/NAMPT role in the pathogenesis of atopic dermatitis

Arroyo et al. (15) found that visfatin/NAMPT, an enzyme crucial for NAD+ production, was significantly elevated in the spinous layer of the skin in AD patients. Some basal keratinocytes and dermal cells also showed increased visfatin/NAMPT levels. Moreover, patients with higher levels of visfatin/NAMPT and poly(ADP-ribose) (PAR) also exhibited increased levels of proliferating cell nuclear antigen (PCNA), a marker of cell proliferation. This suggests a link between visfatin/NAMPT activity and increased cell growth in AD skin (15) (**Table 3**).

**Table 3:** Summary of the studies on visfatin/NAMPT role in atopic dermatitis.

Author	Year	Population	Key observation
Visfatin/NAMPT in atopic dermatitis			
Arroyo et al. (15)	2023	N1–10 healthy people N2–6 patients with AD	Visfatin/NAMPT, an enzyme crucial for NAD+ production, was significantly elevated in the spinous layer of the skin in AD patients.
Suga et al. (50)	2013	N1–42 healthy people N2–40 patients with AD	In AD patients, visfatin/NAMPT levels correlated with eosinophil counts and besides it was correlated with itch severity and levels of certain inflammatory molecules.
Machura et al. (51)	2013	N1–46 healthy people N2–27 patients with AD	A strong positive correlation was found between visfatin/NAMPT concentration and triglyceride levels in children dealing with AD.

PAR, poly(ADP-ribose); PCNA, proliferating cell nuclear antigen; VAS, visual analogue scale; CCL11, C-C motif chemokine 11; CCL26, C-C motif chemokine 26.

Suga et al. (50) showed that serum visfatin/NAMPT levels were significantly higher in patients with AD compared to healthy individuals. In AD patients, visfatin/NAMPT levels correlated with eosinophil counts, moreover, it was related to itch severity (measured by visual analogue scale (VAS)) and levels of certain inflammatory molecules (C-C motif chemokine 11 (CCL11) and C-C motif chemokine 26 (CCL26)). In addition, visfatin/NAMPT levels increased with AD severity (mild, moderate, severe). An adult-onset AD also showed significantly higher visfatin/NAMPT levels compared to classical childhood-onset AD. In conclusion, an increased visfatin/NAMPT level could be a biomarker for disease severity in AD (50) (**Table 3**).

In contrast to the abovementioned studies, Machura et al. (51) observed that serum visfatin/NAMPT level was significantly lower in children with AD compared to healthy controls. The ratio of visfatin/NAMPT levels to BMI was also lower in children with AD. However, visfatin/NAMPT levels were similar between normal-weight and obese children with AD. A strong positive correlation was found between visfatin/NAMPT concentration and triglyceride levels in children dealing with AD. The study suggests that visfatin/NAMPT levels could potentially be used to distinguish children with AD from healthy children (51) (**Table 3**).

### 2.4 Therapeutic potential

#### 2.4.1 Elafin

Elafin, a serine protease inhibitor, holds clinical value in several areas due to its anti-inflammatory and anti-proteolytic properties. It is being investigated as a potential biomarker and therapeutic target in various inflammatory and neoplastic diseases, including inflammatory bowel disease (IBD), acute graft-versus-host disease (aGVHD), and cancers. Phase II trials are underway to investigate the therapeutic effects of elafin on post-operative inflammation and morbidity after major surgeries, such as esophagectomy (for esophageal cancer) (52), coronary artery bypass surgery (53) and pulmonary arterial hypertension (54).

#### 2.4.2 Chemerin

Chemerin has emerged as a potential biomarker with clinical value in various diseases. It plays a role in inflammation, metabolism, and immune responses, and its levels in the body can be associated with conditions like obesity, cardiovascular disease, and certain cancers. Preclinical research in mice has explored modulating the chemerin/CMKLR1 axis, using chemerin-derived peptides or CMKLR1 inhibitors, for potential treatments for neuropathic pain and allergic airway inflammation. However, there is a lack of clinical trial data in humans (55).

#### 2.4.3 Visfatin/NAMPT

Visfati/NAMPT is an adipokine with potential therapeutic applications, particularly in metabolic disorders and inflammatory conditions. It can influence various cellular processes, including glucose metabolism, inflammation, and angiogenesis. More than 100 patients were treated with CHS-828 (later known as GMX1778), a potent and specific NAD(+) inhibitor (NAMPT), in a phase I clinical trial (56). The drug was administered orally. The most common adverse events were thrombocytopenia and gastrointestinal (GI) effects such as diarrhea, vomiting, and esophagitis (57, 58). While visfatin holds promise as a therapeutic target, further research is needed to fully understand its complex mechanisms of action and to address potential toxicity concerns. Clinical trials are necessary to validate the safety and efficacy of visfatin-targeted therapies.

### 2.5 Conclusions

In summary, the available literature data indicate that elafin, chemerin, and visfatin/NAMPT play an important role in epidermal barrier function and inflammation in AD. Their potential stems from dual pro- and anti-inflammatory properties, suggesting a complex role in regulating the inflammatory balance in this dermatosis. For example, it was shown that chemerin restricts the growth of a variety of skin- associated bacteria but on the other hand promotes immune cells migration to the site of inflammation. Therefore, the usage of substances that can specifically regulate the activity of the studied proteins may offer a potential therapeutic approach for the treatment of AD. Available preclinical and early clinical studies have shown their potential safety and tolerability. However, long-term safety and efficacy need to be established in a larger patient population and reliable methods need to be developed to measure levels of these proteins for diagnostic purposes. If successful, these proteins could offer new therapeutic options for atopic dermatitis, potentially complementing or even surpassing existing treatment options.

### 2.6 Limitations

Although knowledge about chemerin, elafin, and visfatin/NAMPT in AD is growing, the inherent complexity and heterogeneity of the disease, combined with inconsistencies in study methodology, pose serious obstacles to drawing clear, comparable, and reproducible conclusions from scientific studies. There is a lack of uniform research methods and standards. Many studies differ significantly in terms of experimental design, such as *in vitro* experiments, animal models, or small-scale clinical trials, making it difficult to directly compare and analyze the results of different studies. Therefore, larger, longitudinal studies are needed in the future to verify these results and better assess the role of these proteins in the development and progression of AD.
